# Intra- and Inter-individual Variability of microRNA Levels in Human Cerebrospinal Fluid: Critical Implications for Biomarker Discovery

**DOI:** 10.1038/s41598-017-13031-w

**Published:** 2017-10-05

**Authors:** Hyejin Yoon, Krystal C. Belmonte, Tom Kasten, Randall Bateman, Jungsu Kim

**Affiliations:** 1 Department of Neuroscience, Mayo Clinic College of Medicine, Jacksonville, FL 32224 USA; 2 Neurobiology of Disease Graduate Program, Mayo Clinic Graduate School of Biomedical Sciences, Jacksonville, FL 32224 USA; 3 Department of Neurology, Washington University School of Medicine, St. Louis, MO 63110 USA

## Abstract

MicroRNAs are emerging as promising biomarkers for diagnosis of various diseases. Notably, cerebrospinal fluid (CSF) contains microRNAs that may serve as biomarkers for neurological diseases. However, there has been a lack of consistent findings among CSF microRNAs studies. Although such inconsistent results have been attributed to various technical issues, inherent biological variability has not been adequately considered as a confounding factor. To address this critical gap in our understanding of microRNA variability, we evaluated intra-individual variability of microRNAs by measuring their levels in the CSF from healthy individuals at two time points, 0 and 48 hours. Surprisingly, the levels of most microRNAs were stable between the two time points. This suggests that microRNAs in CSF may be a good resource for the identification of biomarkers. However, the levels of 12 microRNAs (miR-19a-3p, miR-19b-3p, miR-23a-3p, miR-25a-3p, miR-99a-5p, miR-101-3p, miR-125b-5p, miR-130a-3p, miR-194-5p, miR-195-5p, miR-223-3p, and miR-451a) were significantly altered during the 48 hours interval. Importantly, miRNAs with variable expression have been identified as biomarkers in previous studies. Our data strongly suggest that these microRNAs may not be reliable biomarkers given their intrinsic variability even within the same individual. Taken together, our results provide a critical baseline resource for future microRNA biomarker studies.

## Introduction

MicroRNAs (miRNAs) are small noncoding RNAs that regulate gene expression by directly binding to their target mRNAs^1^. They have critical roles in development, cellular homeostasis, and the pathogenesis of diseases^2^. Certain miRNAs are selectively secreted to extracellular spaces, depending on cellular physiological conditions^3,4^. Furthermore, secreted miRNAs appear to be protected from degradation in various body biofluids^5–7^. Therefore, miRNAs in biofluids have been suggested as minimally invasive biomarker candidates for many human diseases^8,9^. Among the various body biofluids, cerebrospinal fluid (CSF) has attracted great attention in the neurological disease fields because it may reflect the pathological condition of brain tissue. Covered by a skull and dura, the brain is unique in that it is one of the most difficult organs to access directly. Because CSF has a direct contact with brain tissues and reflects the changes in the local milieu of the brain cells, the CSF proteome has been studied for the biomarker discovery for many neurological diseases, including Alzheimer disease (AD) and Parkinson disease.

In addition to the growing attention to miRNA biomarkers in the cancer field, several groups have identified differentially regulated miRNAs using CSF samples from patients with various neurological diseases. In particular, AD has been extensively studied among the neurodegenerative diseases^10^. Although several studies have identified the differentially expressed miRNAs in the CSF of AD patients, the miRNA profiling results were not well replicated from one cohort to another. Moreover, some studies reported that putative AD biomarkers lack any overlap or even conflict between different research groups. For example, a couple of studies reported that the levels of miR-125b and miR-146a were significantly increased in AD CSF^11,12^. However, another study demonstrated that the levels of those two miRNAs were significantly decreased in AD CSF^13,14^. In addition, others reported that the level of miR-125b was increased but the level of miR-146a was decreased in the CSF of AD patients^15,16^. Moreover, while Lehmann *et al*. reported that let-7b levels were increased in AD CSF^17^, others could not replicate it^18^. Although one study reported miR-27a-3p as a putative biomarker in AD CSF^19^, this finding has not been replicated in other AD CSF study^15^. Obviously, various technical limitations and demographic diversity between sample cohorts may have contributed, in part, to such conflicting results^20–22^. In addition, the intrinsic biological variability may also be responsible for the conflicting data.

Intrinsic variability arises from random biochemical reactions of biological molecules over time^23^. Such stochastic dynamics will have pronounced effect on the level of molecules especially when the level of molecule is low^24^. Thus, intrinsic variability in the level of secreted miRNA is a critical variable to consider in the biomarker discovery and development. If the baseline level of a particular miRNA is affected by minor environmental or biochemical changes over a short period of time, the change in miRNA level may reflect not only the disease-related perturbation but also a simple intrinsic noise in miRNA gene expression. While the longitudinal stability of protein biomarkers has been considered as a critical factor for the reliability of biomarkers^25^, intra-individual variability of miRNA level has not been studied yet. To address this critical gap in our understanding of miRNA expression variability, we performed miRNA profiling experiments with CSF samples collected from healthy young adults at two time points and evaluated the intra- and inter-individual variability of miRNA levels. Surprisingly, we found that the levels of most miRNAs in CSF are not variable during 48 hours interval. We also identified a few miRNAs whose expression levels were altered during the 48 hours. Our results provide a valuable resource for miRNA biomarker studies using human CSF samples.

## Results

### Experimental design and development of detection assays

**Figure 1:**
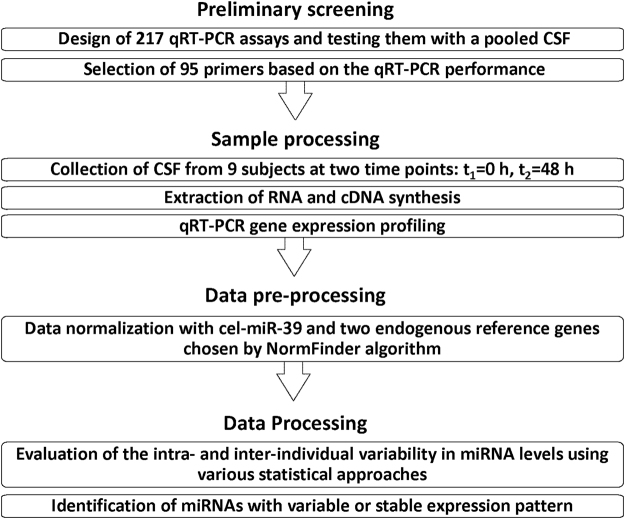
Experimental scheme to determine the intra- and inter-individual variability of miRNA expression levels. First, preliminary screening was performed with a pooled CSF to test the technical quality of 217 miRNA qRT-PCR assays. We selected 95 miRNAs among 217 miRNA assay candidates based on cycle threshold, melting temperature curve, and technical variability. During the sample processing step, CSF samples were collected from nine healthy, young participants at two different time points, 0 and 48 hours. Total RNA was extracted after we spiked in exogenous cel-miR-39 for normalization. To measure miRNA levels, qRT-PCR experiments were performed in duplicate and then data were normalized with reference genes (miR-1246 and miR-374b-5p) and spiked cel-miR-39 internal control. Based on the statistical analyses, the intra- and inter-individual variability in miRNA levels were determined. CSF: cerebrospinal fluid, qRT-PCR; quantitative real-time polymerase chain reaction.

To evaluate the intra- and inter-individual variability of miRNA levels, we performed miRNA expression profiling using human CSF samples. A brief experimental scheme is shown in Fig. 1. For our preliminary screening, we designed 217 miRNA detection assays for quantitative real-time polymerase chain reaction (qRT-PCR). We selected miRNA candidates based on the results from previously reported CSF miRNA studies^26–28^. The total RNAs extracted from 50 randomly selected human CSF samples were pooled and subjected to a qRT-PCR performance test. Based on the quality of qRT-PCR assay performance data, we selected 95 miRNA detection assays based on the following criteria. First, the average cycle threshold (Ct) value from technical duplication must be lower than 36 (with a fixed fluorescence threshold 0.2). Second, the qRT-PCR product must show only a single melting temperature (Tm) curve to ensure the specificity of assay. Third, no signal from a primer dimer could be observed. Fourth, the standard deviation (SD) between technical replications must be less than 0.35. For most miRNA assays, we used the mature miRNA sequences as our forward primer. When such primers generated unreliable data, we extensively modified the primer sequences according to the designing guidelines listed in the Materials and Methods (Supplementary Table S1).

Following the qRT-PCR quality control experiments, CSFs were collected from nine healthy, young participants at two different time points (0 and 48 hours) by lumbar puncture and though a connected cannula. The demographic information of the research participants is summarized in Supplementary Table S2. When determining a time interval between the two time points, we considered two factors that may contribute to the alteration of miRNA levels in CSF, inflammation and circadian rhythm. Inflammation processes have been reported to induce miRNA alteration in serum and CSF^29^. In addition, the levels of some miRNAs in serum fluctuate daily according to circadian rhythm^30^. These results suggest that the levels of some miRNAs in CSF might also be affected by circadian rhythm. Therefore, we selected a time interval of 48 hours to minimize any effect of an acute immune response after lumbar puncture procedures and to rule out any effect of circadian rhythm. Furthermore, to control the diets and physical activities of the study participants, all participants stayed in the clinic before and during the CSF collections.

Sample processing can be described briefly as follows. First, we added a polyacryl carrier to the CSF samples to increase the RNA extraction efficiency. To minimize the technical variability during RNA extraction and cDNA synthesis, we spiked a 200 fmol of synthetic *Caenorhabditis elegans* miRNA (cel-miR-39) into each CSF sample. Because the level of cel-miR-39 detected in qRT-PCR will reflect any technical variability during RNA processing step, it was used as one of the normalization genes. To rule out any plate-to-plate variability, we designed a qRT-PCR layout to compare all CSF samples in one plate with only one miRNA detection assay. All qRT-PCR amplifications were performed with technical duplications. The Ct values used to draw a conclusion were the mean Ct values lower than 36 and had a low SD (<0.25) between technical replications. As a result, we found that 83 miRNAs could be reliably detected in human CSF (Supplementary Table S1).

**Figure 2:**
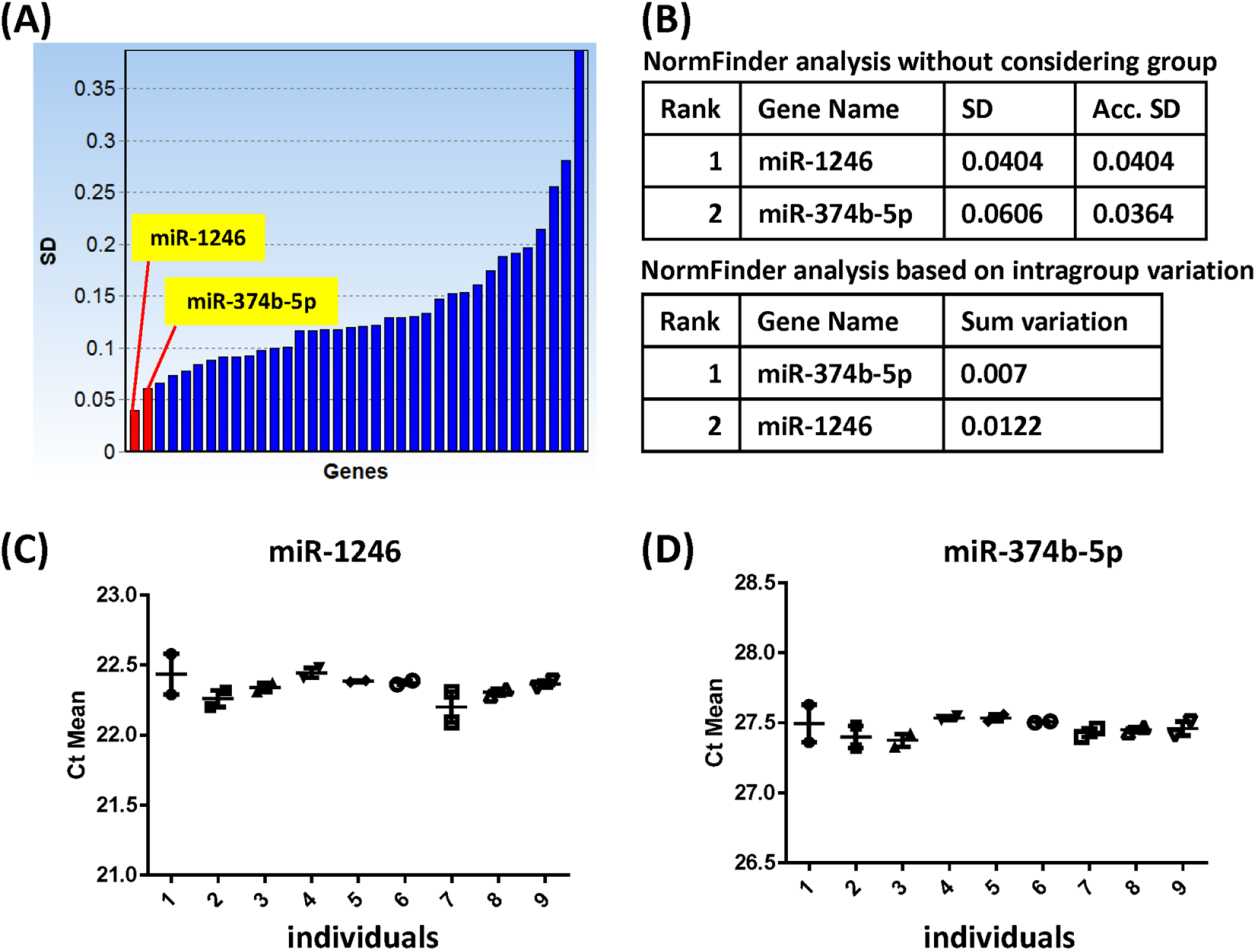
Selection of two reference genes using NormFinder algorithm. (**A**) The SD of miRNA expression levels was calculated. Each bar on the x-axis depicts a miRNA. (**B**) Each table shows the reference gene sets selected by the NormFinder algorithm with two different calculating options. The samples were divided into two groups: the samples collected at time point 0 and those collected after 48 hours. Intra- and inter-group variability were represented as accumulated standard deviation (Acc. SD) or summary of variation (Sum variation), respectively. The same two miRNAs were identified as the most consistent gene sets with or without considering groups. (**C**,**D**) The average Ct values of two reference genes selected by NormFinder algorithm. The x-axis shows each individual participant and the y-axis shows the average Ct value at time points 0 and 48 hours for each individual. Between the two time points, both reference genes exhibited similar Ct values and patterns. Ct; cycle threshold, SD; standard deviation.

The selection of an appropriate normalization method was critical for this study because the RNA concentration in CSF was too low to be reliably detected by spectrophotometers. Thus, the raw Ct values acquired from the qRT-PCR were normalized by the spiked-in cel-miR-39 and two endogenous reference genes. The two endogenous references, miR-1246 and miR-374b-5p, were empirically selected based on the NormFinder algorithm (Fig. 2). The NormFinder algorithm considers intra- and inter-group variability to identify the best combination of reference genes^31^. Here, we assigned two groups to our raw Ct values, the 0-hour and 48-hour groups, according to the time point of CSF collection. Figure 2A shows the SD of miRNA levels calculated by NormFinder algorithm. miR-1246 and miR-374b-5p were identified as the two miRNAs with the lowest variability across all samples without considering the designated groups (Fig. 2B). The same two miRNAs were also identified as the most consistent miRNAs within the designated groups (Fig. 2B). Therefore, we selected both miR-1246 and miR-374b-5p as the best pair of reference genes. Both miRNAs exhibited a similar expression pattern with small Ct differences between 0 and 48 hours (Fig. 2C and D). To determine the variability in a large-scale analysis, we used unique approaches using three different criteria; principal component analysis (PCA), SD of log_2_ relative quantification values (RQV), and Ct-Ct correlation^32–34^. Each approach has its own advantages and disadvantages, which are discussed below.

### Evaluation of miRNA level variability by PCA

**Figure 3:**
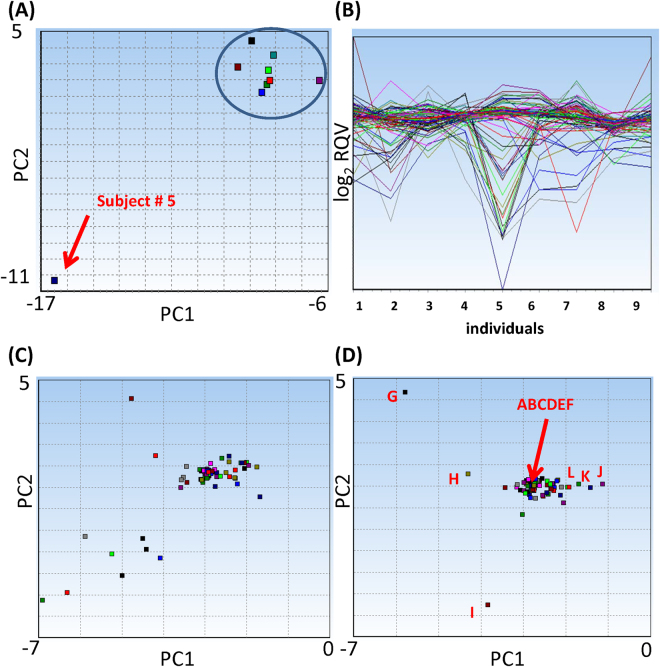
Intra- and inter-individual variability of miRNAs determined by PCA. (**A**) PCA plot showing each individual participant. The variable representing subject #5 is dispersed from the cluster of other eight participants. (**B**) A 2-D line plot depicts each individual on the x-axis and the log_2_ RQV of each miRNA is displayed on the y-axis. (**C**,**D**) PCA plots showing each miRNA before (**C**) and after (**D**) the exclusion of subject #5. Excluding subject #5 decreased the number and distance of miRNAs that were outside of the central cluster. The labels (“A”–“L”) shown in Fig. 3D correspond to miRNAs listed in Fig. 4A–L. The PC scores in Fig. 3A,C, and D are PC1 = 73.92%, PC2 = 88.68% for Fig. 3A; PC1 = 73.92%, PC2 = 88.68% for Fig. 3C; PC1 = 82.23%, PC2 = 89.81% for Fig. 3D, respectively. Log_2_ RQV; log_2_ relative quantification value, PCA; principal component analysis.

PCA converts multiple sets of variables into a decreased dimensional value while retaining most of the information in the original data. Because complex variances in the data sets are transformed into simpler x, y, and z values, PCA is a useful data analysis method to identify common patterns in a large data set by simply comparing the location of the newly generated data points in a graph^32^. Therefore, we applied the PCA method to visualize any difference or similarity in miRNA expression patterns between miRNAs or individual subjects. An outlier in the PCA plot indicates that a particular individual or miRNA is different from the closely clustered others. We determined the variability of miRNA expression level using PCA plot (Fig. 3). First, we analyzed the miRNA expression pattern of each individual to determine if there was any outlier individual with high inter-individual variability (Fig. 3A). In the PCA plot, each dot represents an individual subject. All individuals were grouped together in the plot, except the subject #5. Because a single variable in the PCA plot represents the data from all profiled miRNAs of an individual, the clustering indicates the similarity of miRNA expression patterns between individuals. Conversely, the distinct separation of the subject #5 from the group indicates that this individual had a different miRNA expression pattern compared to other participants. To further investigate the miRNA expression pattern of each individual, we plotted a 2-D line plot of all miRNAs with their corresponding fold changes during the 48-hour interval in log_2_ scale (Fig. 3B). Each miRNA was represented as a different colored line and was plotted across all individuals, showing corresponding fold changes in each individual’s miRNA expression levels. As expected from the PCA analysis, the subject #5 showed dramatic variability in the multiple miRNA expression levels compared to the other subjects. We then generated the PCA plots of each miRNA for all participants to identify the miRNAs with variable expression levels (Fig. 3C). In this plot, each data point represented a single miRNA based on its expression variability from all individuals. A relatively tight clustering of miRNA data points was observed in the center of the PCA plot, while approximately 10 miRNAs were dispersed from the cluster. Because the subject #5 was solely responsible for the high variability in miRNA levels, we excluded this participant from the plot to avoid the false-positive variability driven by this #5 outlier individual. As shown in Fig. 3D, removing the subject #5 dramatically decreased the number of outlier miRNAs. Because the subject #5 was a clear outlier (Fig. 3A), we also excluded the data obtained from this subject in our subsequent analyses, unless otherwise stated.

**Figure 4:**
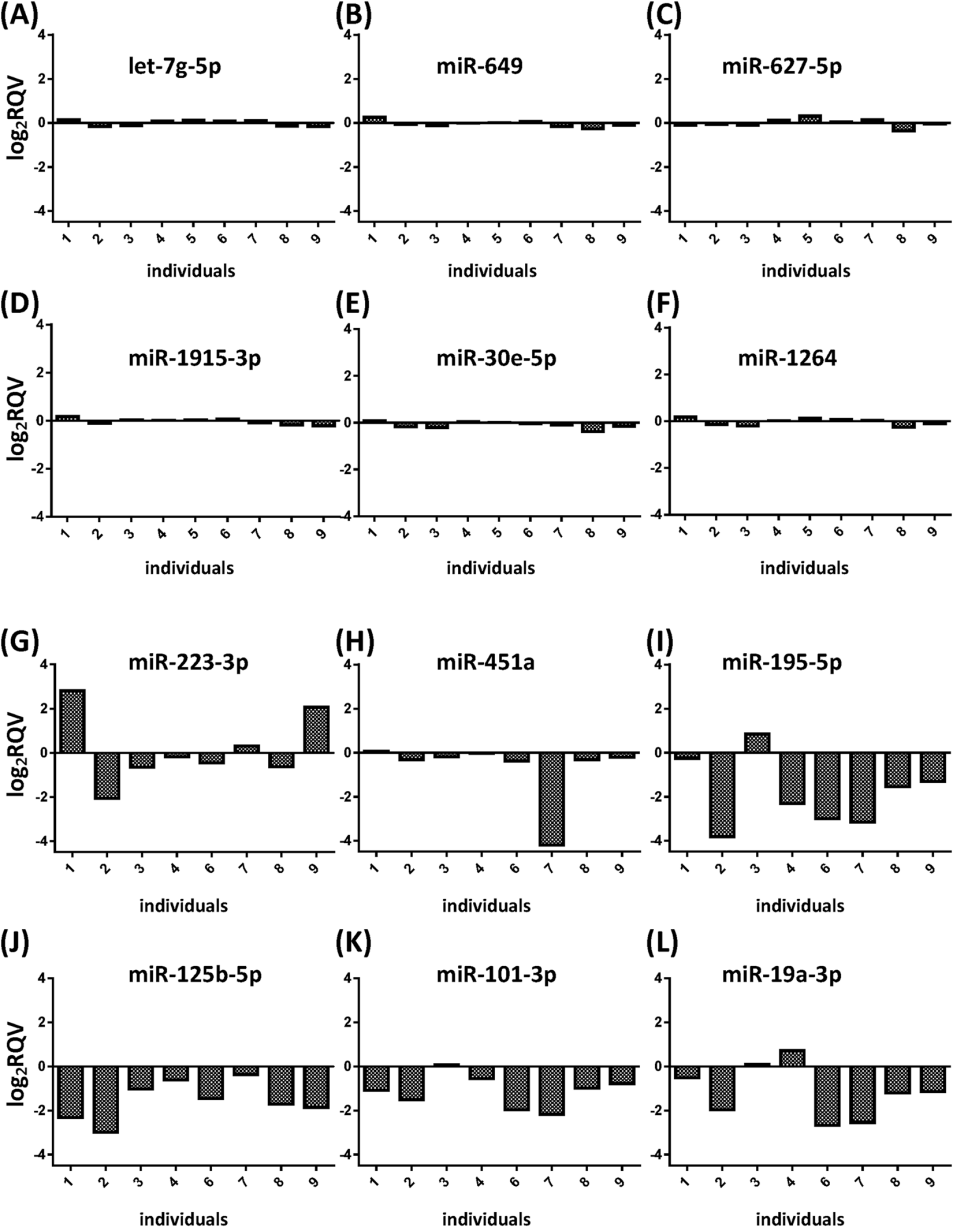
Examples of specific miRNAs with low and high variability identified by PCA. The changes in miRNA expression levels at 48 hours compared to 0 hours are shown in log_2_ values. X-axis depicts each individual and y-axis is log_2_ RQV. The labels correspond with the plot in Fig. 3D. (**A**–**F**) Examples of stable miRNAs. Six miRNAs were selected from the center of the cluster in Fig. 3D. (**G**–**L**) Examples of variable miRNAs. Six miRNAs were selected as examples of miRNAs that were outside of the cluster in Fig. 3D. The graph shows log_2_ RQV of miRNA from each individual. Subject #5 was excluded in Fig. G–L, because the PCA for Fig. 3D was calculated after removing this outlier. Log_2_ RQV; Log_2_ relative quantification value, PCA; principal component analysis.

To further evaluate the variability of miRNA expression level, we selected several miRNAs that were clustered closely or dispersed as outliers from Fig. 3D. The selected miRNAs were indicated as “A”–“L” in Fig. 3D, and their respective log_2_ RQVs were plotted in Fig. 4. The log_2_ RQVs of the selected miRNAs are the relative expression levels of miRNA at the 48-hour time point compared to 0-hour time point. The alphabet indicators (A-L) shown in Fig. 3D match with the labels in Fig. 4A–L. Figure 4A–F show the log_2_ RQVs of stable miRNAs randomly selected from the center of the cluster in the Fig. 3D. These miRNAs exhibited very low intra- and inter-individual variability across all individuals during the 48-hour interval. Because the miRNAs “G”–“L” in Fig. 3D were not clustered with the vast majority of miRNAs in the center of the PCA plot, we hypothesized that these outlier miRNAs will have variable expression levels among the subjects. To test this hypothesis, we calculated their log_2_ RQVs (Fig. 4G–L). The log_2_ RQVs of miR-223-3p varied among participants with variable effect size and direction. The level of miR-223-3p was increased in the subject #1 and #9, but decreased in the subject #2 (Fig. 4G). These results indicate that miR-223-3p has high intra- and inter-individual variability. miR-451a was also classified as a variable miRNA in the PCA plot (labeled as “H” in the Fig. 3D). Although miR-451a was stably expressed across most individuals, the inter-individual variability of this miRNA in Fig. 3D appeared to be high due to the variability in the subject #7 (Fig. 4H). Conversely, miR-195-5p, miR-125b-5p, miR-101-3p, and miR-19a-3p exhibited a consistent decrease in their levels at the 48-hour time point compared to the 0-hour time point across most individuals (Fig. 4I–L). Therefore, miR-195-5p, miR-125b-5p, miR-101-3p, and miR-19a-3p were identified as highly intra-variable miRNAs. Taken together, our results demonstrate that PCA plot is a useful tool to identify the variable miRNAs and we identified several miRNAs that showed short term intra- or inter-individual variability in their expression levels.

### Determination of miRNA level variability using SD of log_2_ RQV

**Figure 5:**
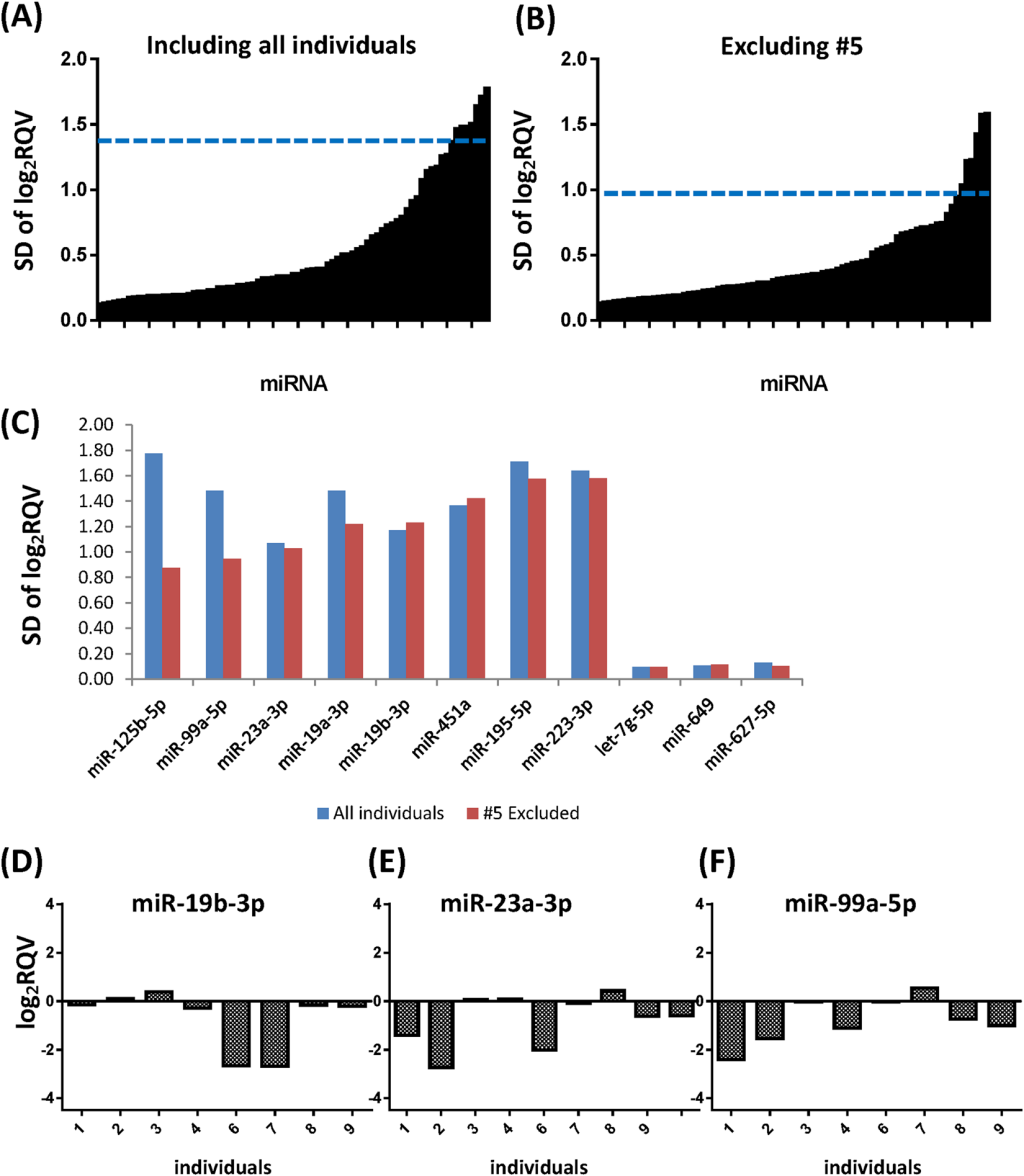
The SD of miRNA levels between individuals indicates inter-variability of miRNAs. (**A**) Inter-variability of miRNAs, including subject #5. The x-axis indicates each miRNA and the y-axis is the SD of expression level change at the 48-hour time point compared to the 0-hour time point. (**B**) Inter-variability of miRNA, excluding subject #5. The inter-variability was decreased after the exclusion of subject #5. The blue line indicates the top 10^th^ percentile of highest miRNA variability. miRNAs above this 10^th^ percentile were selected as high inter-variable miRNAs. (**C**) Direct comparison of the SDs between the stable miRNAs and highly inter-variable miRNAs were shown in Fig. 4. The SD of log_2_ RQV, when all individuals were considered, is shown in blue. The SD of log_2_ RQV, after the exclusion of subject #5, is plotted in red. (**D**–**F**) The analysis based on the SD of log_2_ RQV identified three additional variably expressed miRNAs. Log_2_ RQV of the variable miRNAs was plotted for each individual. Log_2_ RQV; Log_2_ relative quantification value, SD; standard deviation.

Although PCA clustering is an efficient method to determine the variability among miRNAs and individuals, PCA analysis alone cannot identify all variable miRNAs due to its inherent limitation. High inter-individual variability in miRNA level could not be detected in the PCA analysis if the level of a certain miRNA is increased in some participants while decreased to a similar extent in other participants. In that case, the overall variability of this miRNA level will appear to be less in the PCA plot, because the net effect of each alteration may cancel out each other in the PCA analysis. To overcome this critical limitation, we calculated the SD of miRNA levels between individuals to identify additional variable miRNAs (Fig. 5). With this method, a miRNA up-regulated in one individual but down-regulated in another individual would be efficiently distinguished due to its high SD of log_2_ RQV. While a higher or lower RQV itself depicts the intra-individual variability during the 48-hour interval, the high SD of log_2_ RQV between individuals reflects the high inter-individual variability. Determining the variable miRNAs using the SD of log_2_ RQV alone would fail to identify other variable miRNAs when participants’ miRNA expression levels are all decreased or all increased. Therefore, the combination of PCA plot and SD enables us to determine the intra- and inter-individual variability more effectively.

First, we plotted the SD of log_2_ RQV for all individuals (Fig. 5A). Because the subject #5 had a much higher variability in miRNA levels compared to other participants (Fig. 3A), we also analyzed the SDs after excluding the subject #5 (Fig. 5B). The SD reflecting inter-individual variability decreased dramatically when the subject #5 was excluded. We defined variable miRNAs using the cutoff value of the 10^th^ highest percentile of SDs to identify additional miRNAs with high inter-individual variability. The comparison of SDs between the variable miRNAs and stable miRNAs is shown in Fig. 5C. In addition to the variable miRNAs listed in Fig. 4, we found that the expression levels of miR-19b-3p, miR-23a-3p, and miR-99a-5p were also variable by using the SD of log_2_ RQV. The log_2_ RQV plots of each participant are shown in Fig. 5D–F. As expected, high inter-variability between individuals was observed for those miRNAs. The expression levels of those miRNAs were not altered in some individuals, but they were dramatically decreased in other individuals.

### Assessment of miRNA level variability by Ct-Ct correlation

**Figure 6:**
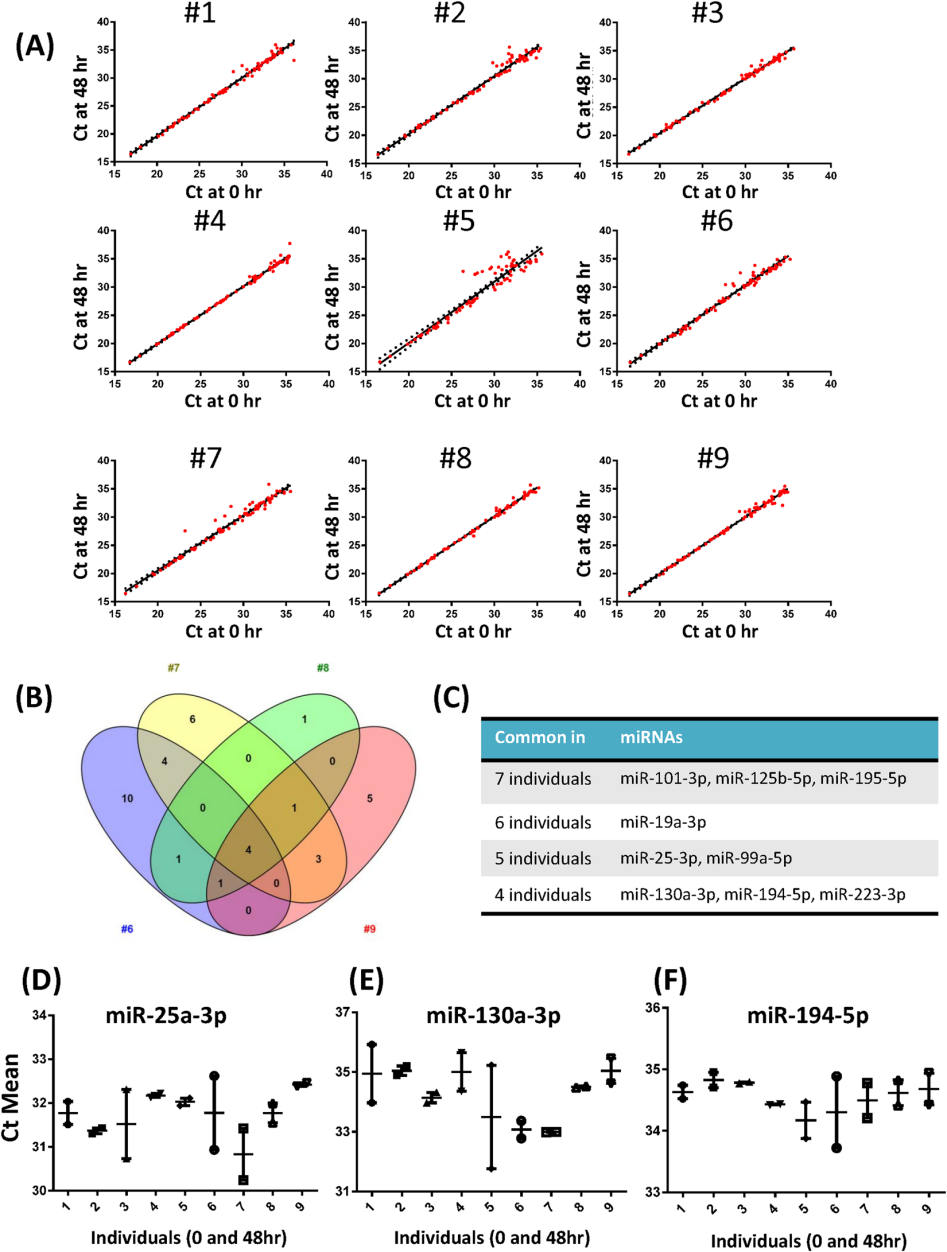
The correlation of Ct values between two time points identifies the intra- and inter-individually variable miRNAs. (**A**) Correlation curves of Ct values at time points 0 and 48 hours. The best fit line with linear regression is shown in black. Each data point represents a single miRNA. miRNAs outside of the 95% confidence interval were defined to be variable. (**B**) The Venn diagram shows the number of variable miRNAs observed commonly among subject #6, #7, #8, and #9. (**C**) A list of highly variable miRNAs commonly observed in the multiple individuals. The miRNAs on the right column were found to be variable in the number of participants shown in the left column. (**D**–**F**) The average Ct value of the variable miRNAs was plotted for each individual at time points 0 and 48 hours. The analysis based on the correlation of Ct values identified three additional variably expressed miRNAs. Ct; cycle threshold.

To determine whether there was any correlation between the Ct values at the two time points, we plotted the linear regression curves with a 95% confidence interval (CI) (Fig. 6A). The extent of the correlations was compared between the two time points within the same individual and between individuals. The *R* ^2^ values from subject #1 to #9 were 0.98, 0.98, 0.99, 0.99, 0.91, 0.98, 0.97, 0.99, and 0.99, respectively. We defined a miRNA to be variable when it fell outside of the 95% interval. For example, the numbers of variable miRNAs commonly found in four participants (subject #6 to #9) are listed in Fig. 6B. Because of the high variability in multiple miRNAs, subject #5 was excluded from further analysis. Based on the 95% CI criteria, we identified multiple miRNAs that had significantly variable expression in more than four individuals (Fig. 6C). Interestingly, there were no miRNA whose expression level was variable across all 8 individuals. By using this Ct-Ct correlation analysis, miR-25-3p, miR-130a-3p, and miR-194-5p were additionally identified as intra-individually variable miRNAs. Their mean Ct values are shown for the time point 0 and 48 hours (Fig. 6D–F).

### Validation of technical reliability in miRNA measurement

**Figure 7:**
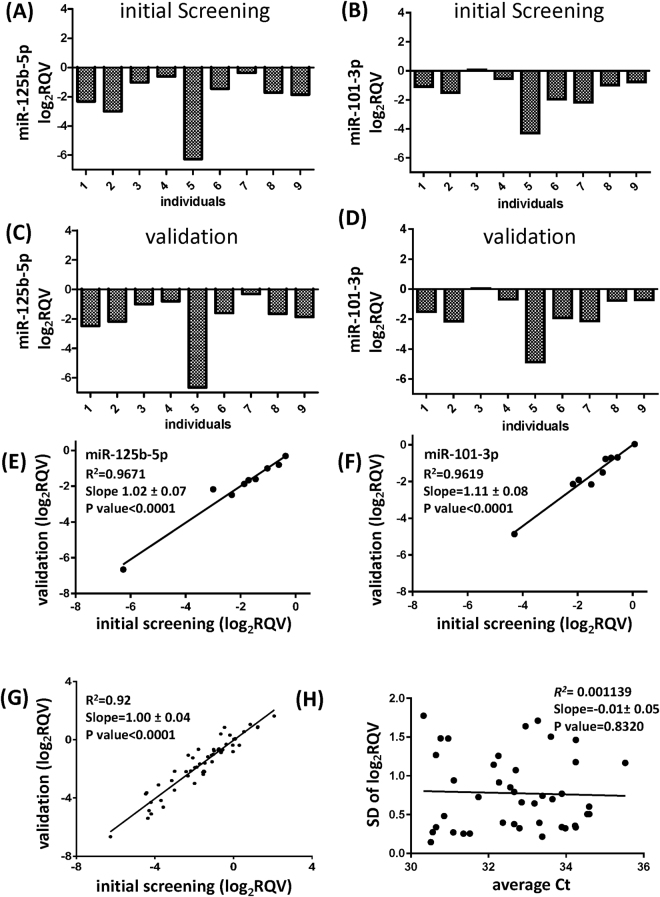
Validation of technical reliability in miRNA measurement. (**A**,**B**) The individual expression levels of miR-125b-5p and miR-101-3p that were identified as highly variable during the initial screening. (**C**,**D**) The levels of variably expressed miRNAs shown in A and B were measured again by an independent researcher. (**E**,**F**) The correlation curve between the initial screening and the replication experiments for miR-125b-5p (**E**) and miR-101-3p (**F**) measurements. Log_2_ RQVs from two independent qRT-PCR assays are significantly correlated. (**G**) Summary of the correlation between the initial screening results and the validation qRT-PCR assay results of 12 highly variable or stable miRNAs. Log_2_ RQVs from two independent qRT-PCR assays are strongly correlated. (**H**) Correlation curve between the Ct values and the SD of log_2_ RQV for miRNAs with Ct values higher than 30. The linear regression curves indicate that there is no correlation between the Ct value and the SD of log_2_ RQV. Ct; cycle threshold, log_2_ RQV; log_2_ relative quantification value, qRT-PCR; quantitative real-time polymerase chain reaction, SD; standard deviation.

When determining the inherent biological variability of gene expression, it is important to validate that the high variability of a particular miRNA was not simply due to our technical mistake. To address this critical concern in quality control, two different individuals repeated the qRT-PCR assays with technical duplication. During initial miRNA expression profiling, miR-125b-5p and miR-101-3p showed dramatically variable expression patterns (Fig. 7A and B). When the experiments were later repeated, we reproduced almost identical data (Fig. 7C and D). In particular, the high variability seen in subject #5 was replicated well during the validation study (Fig. 7C and D). The linear regression analysis indicated that the initial screening and the validated qRT-PCR data were significantly correlated (Fig. 7E and F). To additionally validate our variability data, we expanded the replication experiments to 12 miRNAs with highly variable or stable expression. Using the same linear regression analysis method, we found that there was a strong correlation between the two independent replication experiments (Fig. 7G). Therefore, these results demonstrate that the variability of miRNA levels was not due to any experimental artifact or our technical error.

Even without any technical mistake, any qRT-PCR assay with low signal intensity is likely to generate variable results. Therefore, we analyzed whether there was any correlation between weak qRT-PCR signals and high variability. A Ct value of 30 was used as a cut off for a weak detection signal. No significant correlation was found between the Ct values and the miRNA variability, when variability was assessed by the SD of log_2_ RQV (Fig. 7H).

In conclusion, we found that the levels of most miRNAs in CSF were not variable during a 48-hour window. This result suggests that miRNAs in CSF may be a reliable resource to find biomarkers for neurological diseases. However, we identified 12 miRNAs that had high intra- or inter-individual variability. Table 1 summarizes all variable miRNAs found by three different approaches, PCA, high SD, or Ct-Ct correlation.

**Table 1:** The list of miRNAs with highly variable expression level.

miRNA	Dispersed dots on PCA	10 percentile with high SD	Outlier from Ct-Ct correlation	Reported to be a biomarker
miR-19a-3p	*	*	*	(44)
miR-19b-3p		*		(44)
miR-23a-3p		*		(42)
miR-25-3p			*	
miR-99a-5p		*	*	
miR-101-3p	*		*	
miR-125b-5p	*	*	*	(11–16,42)
miR-130a-3p			*	
miR-194-5p			*	
miR-195-5p	*	*	*	(42)
miR-223-3p	*	*	*	(42,43)
miR-451a	*	*		(40)

Based on three different analytical approaches using the principal component analysis, the standard deviation of relative levels and the correlation of cycle threshold values, we identified 12 miRNAs with high intra- or inter-individual variability. From the left, each table displays the results from Figs 4, 5, and 6, respectively. Some of the variable miRNAs have been reported to be putative biomarkers and the related references are listed. Ct; cycle threshold, PCA: principal component analysis.

## Discussion

Although the expression profiles of circulating miRNAs have been extensively studied under various disease conditions, it is noteworthy that there has been a lack of consistent finding and even conflicting data among miRNA biomarker studies^16,20–22^. Such discrepancies have been largely attributed to technical issues, such as a difference in the sample processing and analytical approach. In addition to the technical limitations, inherent physiological characteristics, such as noise in miRNA gene expression over a short period, may also be responsible for such conflicting results. However, the biological variability in miRNA levels has not been thoroughly investigated.

A few miRNAs were reported to fluctuate during circadian rhythm^30^, menstrual cycle^35^ or exercise^36^. Likewise, only a few studies investigated whether miRNA levels fluctuated under certain physiological conditions. However, the intra-individual variability in CSF miRNA levels has not been addressed in the previous studies. This may be largely due to a difficulty of collecting longitudinal CSF samples within a short interval from healthy volunteers. The extent of intra-individual variability in miRNA levels is a critical question that must be answered before establishing any clinically useful biomarkers because miRNAs with intrinsically noisy expression levels are unlikely to be reliable biomarkers.

In this study, we performed miRNA profiling experiments using longitudinal CSF samples from young healthy participants to evaluate the intra- and inter-individual variability in miRNA levels. To minimize any possible confounding environmental factors, such as the participants’ physical activities and diets, we asked all participants to stay in the clinic during the CSF collection time period. Therefore, our CSF sample collection procedure was well designed to evaluate a biological variability in miRNA levels.

We detected a relatively smaller number of miRNAs in CSF, compared to other miRNA profiling studies^19,28^. This result appears to be due to the use of antemortem CSF samples in our experiments. It has been reported that more miRNAs can be detected in postmortem CSF, because CSF can be contaminated by miRNAs released from degenerated brain tissues^19,37^. Because we collected CSF from young, healthy participants, our CSF samples are likely to reflect a more physiological condition than postmortem CSF samples. Although we measured only 95 miRNAs, we believe that those selected 95 miRNA assays will cover most miRNAs reliably detectable in human antemortem CSF. Because miRNAs have strong tissue-specific expression patterns, only approximately 300 miRNAs out of ~2,500 total human miRNAs were detected in human brain tissues, and about 100 miRNAs account for most miRNAs in the brain^38,39^. Therefore, we designed and optimized the miRNA detection assays based on the previous miRNA profiling studies using CSF. Surprisingly, the vast majority of miRNAs were very stable at the 0-hour and 48-hour time points in our study. This finding suggests that miRNAs in CSF can be reliable biomarker candidates, because the constant expression level of a biomarker is critical for its high reliability and sensitivity.

While most miRNAs were very stable, we also identified 12 miRNAs with a high variability in human CSF (Table 1). The intra- and inter-individual variability observed in our profiling experiments may be associated with extrinsic environmental factors or unknown disease conditions. Previous CSF miRNA profiling studies demonstrated that certain conditions may affect the expression levels of miRNAs in the CSF. For example, Wan *et al*. recently reported that 16 miRNAs were significantly up- or down-regulated in the CSF of patients with major depressive disorder compared to healthy controls^40^. Among the down-regulated miRNAs in the patients, miR-451a was significantly decreased, with a fold change of 8.7. Although we identified miR-451a as a variable miRNA in our PCA analysis, this result was mainly caused by the intra-variability of a single participant. In our study, miR-451a exhibited a 16-fold decrease only in subject #7 during our 48 hour study, while the level of miR-451a in other participants remained stable (Fig. 4H). Because we happened to measure 4 other miRNAs among the 16 miRNA biomarkers reported by Wan *et al*. (miR-30a-5p, miR-33a-5p, miR-139-5p, and miR-451a), we further examined the levels of these miRNAs in subject #7. Interestingly, all 4 miRNAs showed the same up- or down-regulation expression pattern as reported by Wan *et al*. Therefore, it is tempting to speculate whether emotional status of subject #7 affected the miRNA expression level in CSF.

More importantly, our results suggest that the intrinsic variability in miRNA levels may explain the conflicting results from other biomarker studies. The level of miR-125b was reported to be significantly increased^11^, decreased^13,14^, or not altered^16^ in the CSF of AD patients. In our experiments, the levels of miR-125b were highly variable even within the same individual during the 48-hour interval in almost 90% of tested participants (Fig. 4J). Such data suggests that the discrepancy between studies may be due to the intrinsic variability of miR-125b itself. In addition, miR-19b-3p in CSF has been reported as a putative diagnostic marker for glioma and primary central nervous system lymphoma^41^. However, our variability analysis indicated that miR-19b-3p levels can be variable within the same individual even within a short 48-hour interval (Fig. 5D). Furthermore, a recent CSF miRNA profiling study reported that nine miRNAs were significantly decreased in patients with fibromyalgia compared to controls^42^. Among the miRNA biomarkers identified in this study, miR-223-3p, miR-195-5p, and miR-125b-5p were found to be highly variable in our study. Taken together, our data suggests that the discrepancy between CSF biomarker studies may be in part due to the intrinsic variability of miRNA itself^43,44^.

In summary, we demonstrated that miRNAs in CSF are reliably detectable and very stable between 0- and 48-hour time points in most healthy individuals. To our knowledge, our large-scale study is the first to provide strong evidence that the basal levels of most miRNAs in CSF are not variable under normal physiological conditions. This finding suggests that miRNAs in CSF can be reliable biomarker candidates for neurological diseases. In addition, we identified a few highly variable miRNAs that may need to be excluded or carefully re-evaluated in future biomarker discovery studies. Furthermore, we provided the list of stable and variable miRNAs in CSF along with the custom-designed qRT-PCR assay details (Supplementary Table S1). Our experimentally validated miRNA detection assays will also help the research community increase the rigor and reproducibility of their studies.

## Materials and Methods

### Ethics

All analyses were performed with de-identified samples. All subjects gave informed consent to participate in this study, which was approved by the ethics committee at Washington University in St. Louis. All experiments were performed in accordance with relevant guidelines and regulations.

### Subjects information and CSF collection

CSF samples were extracted from 9 healthy individuals by lumbar puncture in the L3-L5 intervertebral space at two different time points, 0 and 48 hours, resulting in a total of 18 samples. Subjects stayed in the clinic before and during the CSF collection. All participants received the same diet and food was given in proportion to body weight. The cannula for the CSF collection remained connected during the 48 hour study period to minimize the stress and acute inflammation caused by the lumbar puncture procedures. The collected CSF samples were centrifuged at 2,000 g at 4 °C for 10 minutes, and the supernatants were divided into aliquots and stored at −80 °C until used in the study. The demographic information of the research participants is summarized in Supplementary Table S2.

### Primer design

We designed forward primers for miRNA qRT-PCR assays according to the following guidelines. For most miRNA assays, we used mature miRNA sequences as our forward primer. However, we modified the primer sequences when the miRNA sequence had a too high or low Tm, or if the sequences highly overlapped with other miRNAs at 3’ end region or with any mRNAs. Our optimal Tm was 58.8 °C with ± 5 °C range. When the Tm of the mature miRNA sequence was lower than the optimal range, we added up to a maximum of 6 Adenines to the 3’ end of primer or added Guanine or Cytosine residues to the 5’ end of primer to increase Tm. When the Tm of the mature miRNA sequence was higher than the optimal range, we removed bases from the 5’ end of primer to decrease Tm. The primer should have ideally at least an 18-mer match to the target miRNA sequences and be less than 30-mer in total length.

### RNA extraction and qRT-PCR

Total RNA was extracted from 200 µL CSF per sample using RNAzol RT (Molecular Research Center, Inc.) spiked in with synthetic cel-miR-39 mimics (Insight Genomics) and a polyacryl carrier (Molecular Research Center, Inc). RNAs were reverse transcribed using the Mir-X miRNA First-Strand Synthesis Kit (Clontech) according to the manufacturer’s instructions. qRT-PCR was performed with StepOnePlus Real-Time PCR systems (Thermo Fisher Scientific) using Fast SYBR Green Master Mix (Thermo Fisher Scientific) under the default thermal cycling program. A total of 96 miRNAs including cel-miR-39 were measured from the CSF samples with technical duplicates.

### Normalization of miRNA expression level and downstream data analysis

NormFinder algorithm in the GenEx 5.3.2 software (MultiD Analyses AB) was used to identify the reference genes that had the lowest variability across all samples. To ensure that we performed the NormFinder analysis with only reliable miRNA expression data, we prefiltered the input miRNA list by removing the miRNAs with high SD in the upper second quartile. Then we recalculated the variability with the filtered reference gene candidates using NormFinder algorithm. The samples were divided into two groups, the samples collected at time point 0 and the samples collected after 48 hours. The best combination of two normalization genes was selected, with and without considering the group differences. When group differences were considered, the statistical method evaluated the variability of miRNA levels under the assumption that traits from one group could be different from those of another group. Whereas, when the group differences were not considered, the other statistical approach evaluated the variability with the assumption that all values were from one homogeneous group. The raw Ct values were normalized by spiked-in cel-miR-39 and then two endogenous reference genes selected based on the NormFinder algorithm. After the normalization, we used GenEx 5.3.2 software to calculate the RQV at the 48-hour time point compared to 0-hour time point and presented the RQV as log_2_ fold changes. miRNA stability was determined by the clustering in PCA and by using the SD of log_2_ RQV. The PCA was drawn using GenEx 5.3.2 software. The datasets generated during the current study are available from the corresponding author on reasonable request.

### Statistics

Linear regression, Student *t* test, and 1-way analysis of variance were performed as indicated. All data were analyzed using GraphPad Prism 6.00 for Windows (GraphPad Software, Inc) with the following values considered significant: *P < 0.05, **P < 0.01, ***P < 0.001. All data are shown as mean ± SEM.

## Electronic supplementary material

Supplementary Tables
