# Supplementary material for: Intra- and Inter-individual Variability of microRNA Levels in Human Cerebrospinal Fluid: Critical Implications for Biomarker Discovery
Source: Sci Rep. 2017 Oct 5;7:12720. doi: 10.1038/s41598-017-13031-w (PMC5629256; doi:10.1038/s41598-017-13031-w)
Supplement: Supplementary file 1 — Supplementary Tables [file 41598_2017_13031_MOESM1_ESM.doc]

**Supplementary Information**

# Intra- and Inter-individual Variability of microRNA Levels in Human Cerebrospinal Fluid: Critical Implications for Biomarker Discovery

Hyejin Yoon^1,2^

Krystal C. Belmonte^1^

Tom Kasten^3^

Randall Bateman^3^

Jungsu Kim^1,2,*^

^1^Department of Neuroscience, Mayo Clinic College of Medicine, Jacksonville, FL 32224, USA

^2^Neurobiology of Disease Graduate Program, Mayo Clinic Graduate School of Biomedical Sciences, Jacksonville, FL 32224, USA

^3^Department of Neurology, Washington University School of Medicine, St. Louis, MO 63110, USA

^*^To whom correspondence should be addressed: Jungsu Kim, Department of Neuroscience, Mayo Clinic College of Medicine, 4500 San Pablo Rd S, Jacksonville, FL 32224. Telephone: (904) 953-2652. Fax: (904) 953-7370. Email: [kim.jungsu@mayo.edu](mailto:kim.jungsu@mayo.edu)

**Supplementary Table S1**

| **ID** | **Primer Sequence** | **ID** | **Primer Sequence** |
| --- | --- | --- | --- |
| Cel-miR-39 | TCACCGGGTGTAAATCAGCTTG | miR-25-3p | CATTGCACTTGTCTCGGTCTGA |
| let-7e-5p | TGAGGTAGGAGGTTGTATAGTTAAAAAA | miR-27a-3p | TTCACAGTGGCTAAGTTCCGC |
| let-7f-5p | GGGTGAGGTAGTAGATTGTATAGTTAAAAAA | miR-29a-3p | TAGCACCATCTGAAATCGGTTA |
| let-7g-5p | GGTGAGGTAGTAGTTTGTACAGTTAAAAAA | miR-29b-3p | TAGCACCATTTGAAATCAGTGTT |
| let-7i-5p | TGAGGTAGTAGTTTGTGCTGTT | miR-302e | GGTAAGTGCTTCCATGCTTAAAAAA |
| miR-100-5p | ACCCGTAGATCCGAACTTGTG | miR-30a-5p | TGTAAACATCCTCGACTGGAAG |
| miR-101-3p | CGTACAGTACTGTGATAACTGAAA | miR-30d-5p | TGTAAACATCCCCGACTGGAAG |
| miR-1228-3p | TCACACCTGCCTCGCCCCCCAAAA | miR-30e-5p | TGTAAACATCCTTGACTGGAAGAA |
| miR-124-3p | TAAGGCACGCGGTGAATGCC | miR-3141 | GAGGGCGGGTGGAGGAGGA |
| miR-1246 | AATGGATTTTTGGAGCAGGAAAAA | miR-320a | AAAAGCTGGGTTGAGAGGGCGA |
| miR-1247-5p | ACCCGTCCCGTTCGTCCCCGGA | miR-33a-5p | GTGCATTGTAGTTGCATTGCA |
| miR-125b-5p | TCCCTGAGACCCTAACTTGTGA | miR-34b-3p | GCAATCACTAACTCCACTGCCAT |
| miR-1264 | CAAGTCTTATTTGAGCACCTGTTAAAAA | miR-3611 | GCGCTTGTGAAGAAAGAAATTCTTAAAA |
| miR-1272 | GATGATGATGGCAGCAAATTCTGAAA | miR-3614-5p | CCACTTGGATCTGAAGGCTGCCC |
| miR-1287-5p | TGCTGGATCAGTGGTTCGAGTC | miR-374b-5p | GGGATATAATACAACCTGCTAAGTGAAAA |
| miR-129-5p | CTTTTTGCGGTCTGGGCTTGCAAA | miR-409-3p | GAATGTTGCTCGGTGAACCCCT |
| miR-1302 | GGGTTGGGACATACTTATGCTAAAAAA | miR-423-5p | TGAGGGGCAGAGAGCGAGACTTT |
| miR-130a-3p | CAGTGCAATGTTAAAAGGGCAT | miR-425-5p | AATGACACGATCACTCCCGTTGA |
| miR-136-5p | ACTCCATTTGTTTTGATGATGGA | miR-4286 | ACCCCACTCCTGGTACCAAA |
| miR-139-5p | TCTACAGTGCACGTGTCTCCAGTAA | miR-451a | AAACCGTTACCATTACTGAGTTAAAAAA |
| miR-142-3p | GCTGTAGTGTTTCCTACTTTATGGA | miR-485-5p | AGAGGCTGGCCGTGATGAATTC |
| miR-142-5p | GGCATAAAGTAGAAAGCACTACTAAAAAA | miR-539-5p | GGGAGAAATTATCCTTGGTGTGTAAAAA |
| miR-143-3p | GGTGAGATGAAGCACTGTAGCTC | miR-548h-5p | AAAAGTAATCGCGGTTTTTGTCAAA |
| miR-144-3p | CGGGGGTACAGTATAGATGATGTACT | miR-548l | AAAAGTATTTGCGGGTTTTGTCAAA |
| miR-1470 | GCCCTCCGCCCGTGCACCCCGAAAA | miR-571 | TGAGTTGGCCATCTGAGTGAG |
| miR-150-5p | TCTCCCAACCCTTGTACCAGTG | miR-574-3p | CACGCTCATGCACACACCCACAAA |
| miR-1537-5p | GGAGCTGTAATTAGTCAGTTTTCTAAAA | miR-574-5p | TGAGTGTGTGTGTGTGAGTGTGT |
| miR-16-5p | TAGCAGCACGTAAATATTGGCG | miR-582-5p | GTTACAGTTGTTCAACCAGTTACTAAAAAA |
| miR-1913 | TCTGCCCCCTCCGCTGCTGCCAA | miR-613 | AGGAATGTTCCTTCTTTGCCAAAA |
| miR-1915-3p | CCCCAGGGCGACGCGGCGGGAAAAA | miR-618 | AAACTCTACTTGTCCTTCTGAGTAAAA |
| miR-194-5p | TGTAACAGCAACTCCATGTGGA | miR-627-5p | GGTGAGTCTCTAAGAAAAGAGGAAAAA |
| miR-195-5p | GATAGCAGCACAGAAATATTGGC | miR-633 | GGGCTAATAGTATCTACCACAATAAAAAA |
| miR-19a-3p | TGTGCAAATCTATGCAAAACTGA | miR-636 | TGTGCTTGCTCGTCCCGCCCGCAAAA |
| miR-19b-3p | TGTGCAAATCCATGCAAAACTGA | miR-649 | AAACCTGTGTTGTTCAAGAGTCAAA |
| miR-204-5p | TTCCCTTTGTCATCCTATGCCT | miR-659-3p | CTTGGTTCAGGGAGGGTCCCCA |
| miR-20a-5p | TAAAGTGCTTATAGTGCAGGTAG | miR-671-3p | TCCGGTTCTCAGGGCTCCACC |
| miR-21-5p | CGGTAGCTTATCAGACTGATGTTGA | miR-758-3p | TTTGTGACCTGGTCCACTAACC |
| miR-223-3p | TGTCAGTTTGTCAAATACCCCA | miR-874-3p | CTGCCCTGGCCCGAGGGACCGA |
| miR-22-3p | AAGCTGCCAGTTGAAGAACTGT | miR-9-3p | CGCATAAAGCTAGATAACCGAAAGT |
| miR-23a-3p | GGATCACATTGCCAGGGATTTCC | miR-9-5p | GGTCTTTGGTTATCTAGCTGTATGA |
| miR-23b-3p | GGATCACATTGCCAGGGATTACC | miR-99a-5p | AACCCGTAGATCCGATCTTGTG |

**Supplementary Table S1. Development of the custom reliable qRT-PCR assays for miRNAs.** Of the 96 miRNAs evaluated (including cel-miR-39), 82 miRNAs were detected reliably. The cutoff value to determine reliability was the cycle threshold of less than 36 across all individuals with a tight technical replication (standard deviation between replications <0.35). For most miRNA assays, the sequences of mature miRNAs were initially tested as the forward primer sequences. Primer sequences that were modified from the mature miRNA sequences are indicated in red (added sequence) and blue (deleted sequence) color. In this study, we identified 12 miRNAs with high intra- or inter-individual variability that are highlighted in yellow in the ID tab.

**Supplementary Table S2**

| **Participant #** | **age (37.3 ± 2.92)** | **gender** |
| --- | --- | --- |
| 1 | 34 | F |
| 2 | 22 | F |
| 3 | 46 | M |
| 4 | 46 | F |
| 5 | 27 | F |
| 6 | 41 | F |
| 7 | 47 | F |
| 8 | 36 | F |
| 9 | 37 | F |

**Supplementary Table S2. Demographic information of research participants**. Nine healthy individuals participated in this research. Each participant’s age and sex are shown. The average age is 37.3 years and the standard error of the mean is 2.92. There were no abnormal medical condition or medical history reported before and during the sample collection.
